# Using γ-Ray Polymerization-Induced Assemblies to Synthesize Polydopamine Nanocapsules

**DOI:** 10.3390/polym11111754

**Published:** 2019-10-25

**Authors:** Wenwen Jiang, Xinyue Zhang, Yafei Luan, Rensheng Wang, Hanzhou Liu, Dan Li, Liang Hu

**Affiliations:** 1 School for Radiological and Interdisciplinary Sciences (RAD-X), Collaborative Innovation Center of Radiation Medicine of Jiangsu Higher Education Institutions and Jiangsu Provincial Key Laboratory of Radiation Medicine and Protection, Soochow University, Suzhou 215123, Jiangsu, China; 2 College of Chemistry, Chemical Engineering and Materials Science, Soochow University, Suzhou 215123, Jiangsu, China

**Keywords:** polydopamine nanocapsules, polymer assemblies, radiation polymerization

## Abstract

This work reports a simple and robust strategy for synthesis of polydopamine nanocapsules (PDA NCs). First, polymer assemblies were synthesized by a γ-ray-induced liquid–liquid (H_2_O–acrylate) interface polymerization strategy, in the absence of any surfactants. ^1^H nuclear magnetic resonance analysis and molecular dynamics simulation reveal that the generation of polymer assemblies largely depends on the hydrophilicity of acrylate and gravity of the oligomers at the interface. By virtue of the spherical structure and mechanic stability of the polymer assemblies, PDA NCs are next prepared by the interfacial polymerization of dopamine onto the assemblies, followed by the removal of templates by using ethanol. The polydopamine nanocapsules are shown to load and release ciprofloxacin (CIP, a model drug), such that the CIP-loaded PDA NCs are able to inhibit the growth of Escherichia coli.

## 1. Introduction

Polydopamine (PDA) is one of the most popular biomimetic polymers that can robustly bind to many substrates [1,2,3,4]. Studies of PDA-related materials have increased in the past decade [4,5,6,7]. Among these, PDA capsules have gained great attention because of their unique hollow structures and large surface area [8,9,10,11]. PDA capsules have many advantages over capsules made of other materials [12,13,14,15]. PDA is biocompatible and biodegradable, one of the main components of natural melanin, and is widely distributed in the human body [10,16,17,18,19]. PDA can also be easily postfunctionalized [18,20]. PDA bears catechol and amine groups, enabling PDA to immobilize functional components by secondary reactions [2]. PDA is also hydrophilic; its polar groups are responsible for the high surface energy and hydrophilicity of PDA [21]. Therefore, PDA capsules have been extensively used as adsorbents [22], drug loading/delivery platforms [23], and catalysts [11].

PDA capsules are typically prepared by templating approaches [8,9,10,11]. For instance, dopamine (DA) self-polymerizes onto SiO_2_ particles by oxidative polymerization of DA in a mildly alkaline solution. Subsequently, PDA capsules are obtained by etching the SiO_2_ particles in bio-malignant hydrofluoric acid solution [8,10,11,24,25]. Using this concept, many particles, such as polystyrene [26], CaCO_3_ [27], and curcumin crystals [9], are used as sacrificial cores to construct PDA capsules. However, such hard-templating methodology is severely limited by harsh conditions to remove the templates, especially when chemically sensitive moieties (e.g., biomolecules) are expected to be involved [11]. Alternatively, PDA capsules are prepared using monodisperse dimethyldiethoxysilane emulsion drops as soft templates [8,28]. In this method, DA self-polymerizes onto soft templates. The templates are removed by adding ethanol, forming PDA capsules with a diameter of 400 nm to ~2.4 μm [8]. Alkane-in-water emulsion droplets and tetrahydrofuran-buffer mixture have also been reported as soft templates for the synthesis of PDA capsules with sizes of 200−7.5 μm [29,30,31]. Using this technology, many functional substances, such as quantum dots, nanoparticles, and biomedical drugs, can be loaded [8,28,29]. However, soft templates have limited resource, instinct instability, and their use poses difficulty in removing surfactants. Accordingly, the spherical structure of PDA capsules is often damaged.

**Scheme 1:**
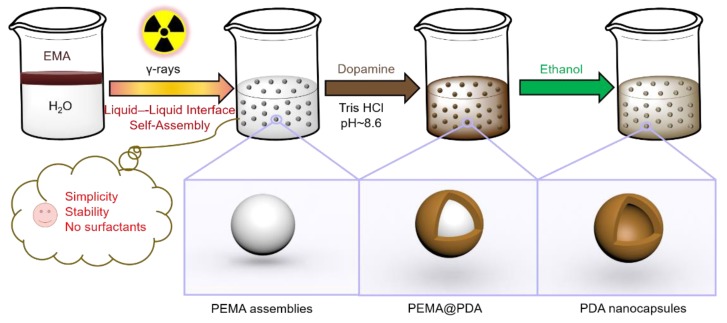
A schematic illustration of the synthesis of PDA NCs by virtue of polymeric assembly templates that were prepared by LLISA polymerization. PDA NCs: polydopamine nanocapsules; LLISA: γ-ray-induced liquid–liquid interface self-assembly; PEMA: Poly(ethyl methacrylate); EMA: ethyl methacrylate.

In this paper, we report a novel strategy for synthesis of a polymer assembly as a soft template to generate PDA nanocapsules (NCs, Scheme 1). Poly(ethyl methacrylate) (PEMA) assemblies were obtained by a γ-ray-induced liquid–liquid interface self-assembly (LLISA) polymerization strategy. The product featured spherical nanostructures (average diameter of 300 ± 80 nm, yield of 88% (determined by 1H nuclear magnetic resonance (NMR) analysis)). PDA NCs were generated by the interfacial polymerization of DA onto polymer assemblies, followed by the removal of templates by using ethanol. Compared with emulsion polymerization and dispersion polymerization [32,33,34], the LLISA polymerization strategy renders the synthesis of polymeric particles (as soft templates) with simplicity, high stability, and no surfactants. Accordingly, the spherical structure of PDA capsules is well retained. Finally, we demonstrated that PDA NCs can load/release a model drug, namely, ciprofloxacin (CIP). The CIP-loaded PDA NCs were shown to inhibit the growth of *Escherichia coli* (*E*. *coli*).

## 2. Materials and Methods

### 2.1. Materials

Methyl methacrylate (MMA, ≥98%), ethyl methacrylate (EMA, ≥98%), sodium chloride (NaCl, ≥99.5%), sodium hydroxide (NaOH, ≥96%), and sodium phosphate monobasic dihydrate (NaH_2_PO_4_·2H_2_O, ≥99%) were supplied by Sinopharm chemical reagent (Suzhou, China). *N*,*N*-dimethylformamide (DMF, ≥99.5%) was purchased from Shanghai Lingfeng Chemical Reagent Co., Ltd. (Shanghai, China). Butyl methacrylate (BMA, ≥99%), hexyl methacrylate (HMA, ≥98%), iso-Octyl methacrylate (OMA, ≥99%), dopamine hydrochloride (DA, ≥98%), and potassium bromide (KBr, ≥99.5%) were purchased from Aladdin (Shanghai, China). Ethanol (≥99.7%) was supplied by Chinasun specialty products Co., Ltd. (Changshu, China). Deuterated chloroform (CDCl_3_, 99.8%, with tetramethylsilane (TMS)) and deuterated acetone ((CD_3_)_2_CO, 99.9%, with TMS) were supplied by Cambridge Isotope Laboratories Inc. (Andover, MA, USA). Oil red O was purchased from Shanghai Zhanyun Chemical Co., Ltd (Shanghai, China). Tryptone, yeast powder, and sodium phosphate dibasic dodecahydrate (Na_2_HPO_4_·12H_2_O, 99%) were supplied by Oxoid (Basingstoke, Hampshire, UK). Ciprofloxacin hydrochloride hydrate (CIP, 98%) was supplied by Energy Chemical (Shanghai, China). *Escherichia coli* (*E.coli*, 25922) was supplied by American type culture collection (Manassas, VA, USA). All these chemicals were used as received. Deionized (DI) water was produced by a Milli-Q Plus system (Millipore Co. Burlington, MA, USA) to have a resistivity of 18.2 MΩ cm.

### 2.2. Synthesis of Polyacrylate Assemblies 

Deionized water (10 mL) was added into a glass vial (20 mL, φ*_inner_* = 26 mm, φ*_outer_* = 31 mm, height = 42 mm), followed by 0.5 mL of acrylates. A water–acrylate interface was generated spontaneously. After purging N_2_ gas for 15 min, the interface was maintained, and the mixture was exposed to γ-rays under room temperature (^60^Co source, 1.85 kGy h^−1^, Nordion Inc., Ottawa, Ontario, Canada) to obtain polymer assemblies. The ^60^Co source (φ = 10 mm, length = 400 mm) was located 35 cm horizontally and 70 cm vertically away from the vial. The conversion of polymerization was determined by ^1^H nuclear magnetic resonance (NMR) analysis.

### 2.3. Synthesis of Polydopamine Nanocapsules (PDA NCs)

Poly(ethyl methacrylate) (PEMA) assemblies (5 mL) and DA (500 mg) were dissolved in 250 mL of Tris HCl buffer solution (~pH 8.6) and stirred at room temperature for 24 h. PDA-coated PEMA (PEMA@PDA) was obtained by centrifugation at 4200 rpm (5 min, 3×). Then, PEMA@PDA assembly was dissolved and stirred in ethanol for 24 h. The mixture was centrifuged at 4200 rpm, (5 min, 3×) to obtain PDA NCs and lyophilized prior to use.

### 2.4. Characterizations

The chemical composition of polymer assembly was investigated by Fourier-transform infrared spectroscopy (FTIR, Thermo iS50, Waltham, MA, USA), ^1^H/^13^C NMR (AVANCEIII HD 400 MHz, Bruker, Switzerland) and X-ray photoelectron spectroscopy (XPS, ESCALAB 250Xi, Thermo fisher, Waltham, MA, USA). Transmission electron microscope with energy-dispersive X-ray spectrometry (TEM-EDS, FEI Tecnai G2 spirit BioTwin, Hillsboro, OR, USA) and scanning electron microscopy (SEM, Hitachi S-4700, Tokyo, Japan) were performed to characterize the morphologies of specimens. The thermodynamic performance was studied by differential scanning calorimetry (DSC, heating rate: 20 K min^−1^, N_2_ gas) and thermogravimetric analysis (TG) (STA449F3 Jupiter, Selb, Deutschland, heating rate: 20 K min^−1^, N_2_ gas).

### 2.5. Molecular Dynamics (MD) Simulation

MD simulations were carried out using the Amorphous Cell module implemented in Materials Studio (Accelyrs, Inc., San Diego, CA, USA) with the Forcite module. The condensed-phase optimization molecular potentials for atomistic simulation studies (COMPASSII) force field were used. The cells contained EMA molecules (×20, layer I), water molecules (×2000, layer III), and PEMA chains (×2, 20 repeated monomer units) at the water–EMA interface (layer II). The pressure (0.0001 GPa) and temperature (300 K) were controlled by the Berendsen and Nose method, respectively. The initial water/PEMA/EMA equilibration state was carried out with isothermal–isobaric (NPT, the number of particles N, the pressure P, and the temperature T of the system are kept constant) ensemble. The MD was further performed with NVT ensemble (the number of particles N, the volume V, and the temperature T of the system are kept constant), with a time step of 1 fs and a total of 200 ps. The electrostatic and van der Waals forces were controlled by the atom-based method (cutoff = 1.25 nm, spline width = 0.1 nm, and buffer width = 0.5 Å). The same procedure was performed for other water/polymer/monomer systems.

The self-diffusion coefficient (D) can be calculated by the Einstein Equation (1):

(1) $$D=\underset{t\to \infty }{\lim}\frac{1}{6t}<r^{2}\left(t\right)>$$

where <*r^2^(t)*> is the ensemble average.

### 2.6. Loading CIP into the PDA NCs

PDA NCs (0.5 mL, 5 mg mL^−1^) and CIP (25 μL, 1 mg mL^−1^) were added to 5 mL of H_2_O, and the resultant mixture was shaken at 60 rpm for 24 h. CIP-loaded PDA NCs (CIP-PDA) were obtained after centrifugation at 10,000 rpm for 10 min to form a pellet and removal of the supernatant. The CIP-PDA was stored in a centrifuge tube prior to use.

### 2.7. Releasing CIP from CIP-PDA

CIP-PDA was dispersed in phosphate-buffered saline (2 mL, 0.01 M PBS) and shaken at 60 rpm at room temperature. The release percentage (*R*%) of CIP can be calculated as follows:

(2) $$R\%=\frac{\Delta I\left(96\right)-\Delta I\left(t\right)}{\Delta I\left(96\right)}\times 100\%$$

where Δ*I*(*t*) is the Δ*I* of CIP in the supernatant at a given time (*t*). Δ*I* = *I*_272_ − *I*_400._ Δ*I* was consistent after 96 h(t). The supernatant was obtained by centrifugation at 10,000 rpm for 10 min. After the UV-Vis measurements, the supernatant was refilled into the original solution, ensuring that the total amount of CIP-PDA solution was consistent.

### 2.8. Antibacterial Evaluation of CIP-PDA

*E.coli* was cultured in 20 mL of Luria Bertani (LB) media at 37 °C under 190 rpm for 18 h. The culture suspension was centrifuged at 4200 rpm for 5 min and then washed with PBS to remove LB and dead bacteria. This step was performed three times. Different concentrations of CIP-PDA (125, 62.5, 31.25, 15.625, 7.8125, 3.90625, 1.953125, and 0.976562 μg mL^−1^) and *E. coli* suspensions (OD_600_ = 0.5) were inoculated in 96-well plates, with a volume of 100 μL. The OD_600_ values of the samples were measured in an interval of 30 min by a multimode microplate reader (Varioskan FLASH, Waltham, MA, USA). The positive and negative controls were also studied.

## 3. Results

**Figure 1:**
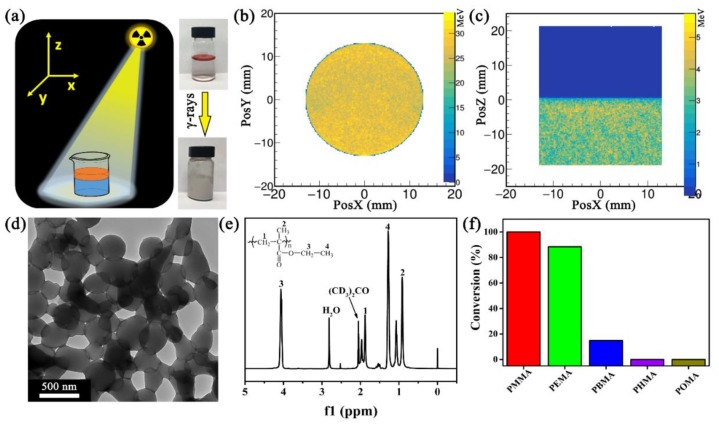
(**a**) Schematic illustration of the experiment setup and the photographs of EMA–H2O mixture (0.5/10 *v*/*v*) before and after γ-ray-induced LLISA polymerization. EMA is dyed by oil red for visualization; GEANT4 (geometry and tracking 4) stimulation of energy deposition on EMA–H_2_O mixture under γ-rays at (**b**) x–y and (**c**) x–z (y = 0) direction; (**d**) TEM and (**e**) ^1^H NMR of PEMA assembly; (**f**) Conversion of different polymers synthesized via γ-ray-induced LLISA polymerization. EMA: ethyl methacrylate.

In a typical experiment, 10 mL of deionized (DI) water and 0.5 mL of ethyl methacrylate (EMA) were added into a 20 mL glass vial. A liquid–liquid (H_2_O–EMA) interface was generated because of the inherent difference in density (1.0 g cm^−3^ for water versus 0.9 g cm^−3^ for EMA) and surface tension (72.8 mN m^−1^ for water vs. 33 mN m^−1^ for EMA)^36^ (Figure 1a). After purging N_2_ gas for 15 min, the mixture was sealed and exposed to γ-rays at a total of 20 kGy (i.e., 20 kJ kg^−1^) under a ^60^Co source. The initial liquid–liquid interface disappeared, while the immiscible H_2_O–EMA mixture transitioned to milky emulsion. In this process, the ^60^Co source delivered homogeneously the energy of the ionizing radiation in the entire system, especially at the H_2_O–EMA interface (Figure 1b,c). This phenomenon facilitated the homogeneous polymerization at the isolated spots. The transmission electron microscopy (TEM) image showed many particles, with an average diameter of 300 ± 80 nm (averaged from 15 particles in Figure 1d). These particles were stable in DI water for months but immediately dissolved in organic solvents (e.g., ethanol, *N,N*-dimethylformamide, and chloroform). In addition, they were also very stable under multiple centrifugation, suggesting their high mechanical stability. Besides, no particles were observed in the absence of H_2_O (data not shown). These results suggest that the use of γ-ray-induced LLISA polymerization led to the generation of the polymer assembly.

γ-Rays of 20 kGy can radiolyse water and EMA, yielding a variety of radicals, including H•, OH•, CH_2_–C•(CH_3_)COOC_2_H_5_, CH•–CH(CH_3_)COOC_2_H_5_, CH_2_=C(CH_3_)COOC_2_H_4_•, and/or CH_2_=C(CH_2_•)COOC_2_H_5_. Therefore, the physicochemical composition of the resultant polymeric assembly should be verified. We first used Fourier transform infrared (FTIR) spectroscopy and ^1^H/^13^C NMR to characterize the products. Figure S1 (in Supplementary Materials) shows that the C–H stretching vibration peaks of CH_3_– and CH_2_– groups occur at 2800–3000 cm^−1^, while the peaks for the C=O and C–O–C groups appear at 1730 and 1150–1260 cm^−1^, respectively. In addition, the ^1^H NMR spectrum in Figure 1e shows two characteristic signals at δ = 4.04 and 1.24 ppm, which can be ascribed to –OC*H*_2_CH_3_ and –OCH_2_C*H*_3_. The area ratio of each proton is 2.00(H_1_):2.94(H_2_):1.99(H_3_):3.06(H_4_), which is an indicator of poly(ethyl methacrylate) (PEMA, 2(H_1_):3(H_2_):2(H_3_):3(H_4_)). Additional data from ^13^C NMR, X-ray photoelectron spectroscopy (XPS), differential scanning calorimetry (DSC), and thermogravimetric (TG) analysis (Figures S2–S4 in Supplementary Materials) further indicate that the PEMA assembly was obtained through γ-ray-induced LLISA polymerization.

To understand the underlying mechanism, we selected a series of acrylates with varying alkane chain lengths (*n*) and determined if the surface tension of acrylate ester influences the generation of polymer assemblies. These monomers included methyl methacrylate (MMA, *n* = 1), EMA (*n* = 2), butyl methacrylate (BMA, *n* = 4), hexyl methacrylate (HMA, *n* = 6), and iso-octyl methacrylate (OMA, *n* = 8). The conversion of polymerization sharply decreased with increasing *n* (Figure 1f). In addition to the PEMA assembly, Figures S5 and S6 (in Supplementary Materials) show that LLISA is a versatile protocol for the synthesis of poly(MMA) (PMMA) and poly(BMA) (PBMA) assemblies. By contrast, neither poly(HMA) (PHMA) nor poly(OMA) (POMA) assembly was obtained, and the corresponding mixture remained transparent.

**Figure 2:**
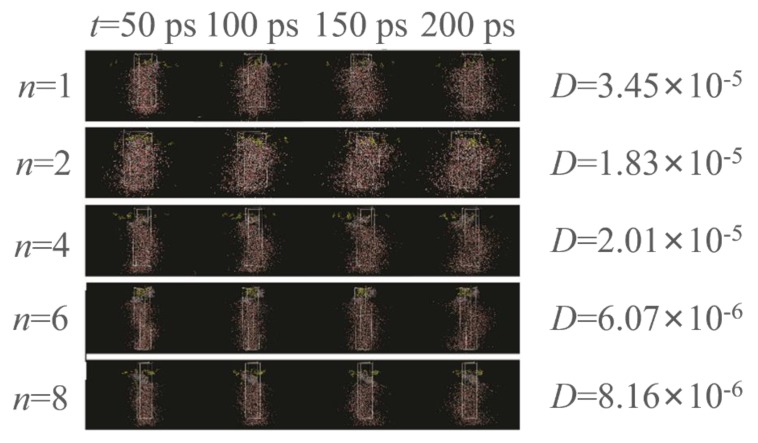
Molecular dynamics (MD) stimulation of the ability of polymers migrating to the water phase.

Molecular dynamics (MD) stimulations were then performed to analyze our hypothesis that relatively more hydrophilic polymers can migrate to the water phase and ultimately generate polymer assemblies (Figure 2). Using PEMA as the example, a cell contained EMA molecules (×20, layer I) and water molecules (2000, layer III). Considering the total cost of simulation time, we set the PEMA chains with 20 repeated monomer units, such that the gravity of the polymer was not considered. The PEMA chains were located at the water–EMA interface as layer II. The initial water/PEMA/EMA equilibration state was performed with NPT isothermal–isobaric ensemble. The MD stimulation was further conducted with the NVT ensemble, with a time step of 1 fs and a total of 200 ps. Self-diffusion coefficients (*D*) were determined by the Einstein equation. The *D* of PMMA, PEMA, and PBMA are higher than those of PHMA and PEHMA. However, the polymer chains at the liquid–liquid interface apparently cannot fully migrate to the water phase driven by the surface tension. Therefore, we infer that exposure of the liquid–liquid mixture to the ionizing radiation under γ-ray promotes the propagation of polymer chains. The oligomers migrated to the H_2_O medium once they grew beyond the solubility limit at the liquid–liquid interface. This phenomenon is possibly due to the hydrophilicity and gravity of the oligomers. As the polymer nuclei are formed and grown in H_2_O, the assembly is generated via entropic precipitation. As *n* increases, the surface tension of acrylates decreases, thereby failing to generate polymer assemblies.

**Figure 3:**
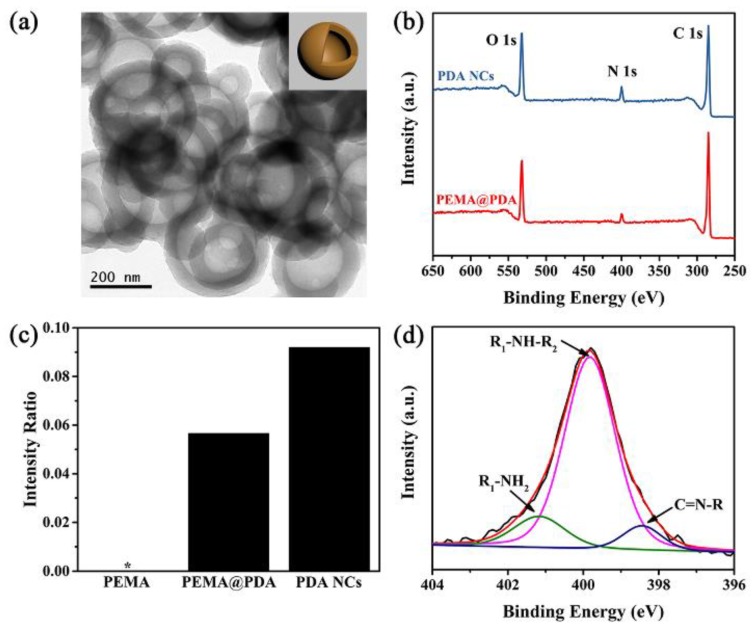
(**a**) TEM image of PDA NCs (inset: a schematic illustration of PDA NCs); (**b**) wide-scan XPS spectra; (**c**) N/C intensity ratio of specimens, and (**d**) N 1s XPS fitting spectrum of PDA NCs.

We moved to synthesize PDA capsules by utilizing the PEMA assemblies as the removal core. Briefly, ~28 wt. % PDA (relative to the PEMA assemblies, Figure S7 in Supplementary Materials) was successfully deposited on the PEMA assembly (PEMA@PDA) via the self-polymerization of DA in Tris HCl solution (pH: ~8.6). The PEMA template was completely removed in ethanol (as shown by ^1^H NMR and FTIR analysis in Figure S8 in Supplementary Materials), generating PDA capsules with a diameter of ~300 nm and thickness of ~45 nm (Figure 3a). The PDA NCs in DI water featured the zeta potential of −14.6 ± 2.5 mV, caused by the deprotonation of the catechol groups when pH is greater than the isoelectric point of PDA (~3.4). The XPS data of PEMA@PDA show the N 1s peak at 400 eV, indicating the successful deposition of PDA on PEMA (Figure 3b and Figure S3 in Supplementary Materials). Accordingly, the intensity of the N 1s signal (relative to the C 1s signal) is enhanced by 0.234 compared with that of PEMA. When the PEMA core was removed, the intensity ratio of N 1s/C 1s further increases to 0.295 (Figure 3c). Specifically, the N 1s peak for PDA NCs can be fitted by R-NH_2_ (401.2 eV), R_1_-NH-R_2_ (399.8 eV), and C=N-R (398.4 eV) because of the diverse chemical compositions of PDA (Figure 3d).

**Figure 4:**
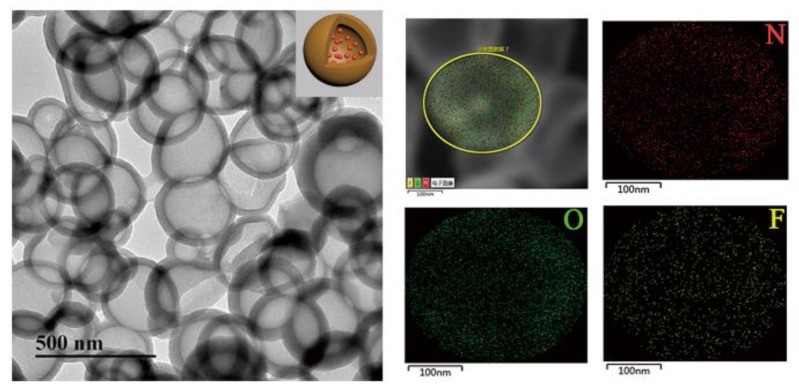
TEM-EDS images of CIP-PDA. (inset: a schematic illustration of CIP-PDA). CIP-PDA: ciprofloxacin (CIP)-loaded PDA NCs.

Finally, we tested the capacity of the PDA NCs to load and release biomolecules of interest. CIP-loaded PDA NCs (or CIP-PDA) were obtained by mixing PDA NCs with CIP (as a model drug) in the solution. The TEM with energy-dispersive X-ray spectrometry (EDS) images in Figure 4 show that the morphology and chemical composition of the PDA NCs were well-retained after loading the CIP. The presence of F indicates that CIP was successfully absorbed by the PDA NCs.

**Figure 5:**
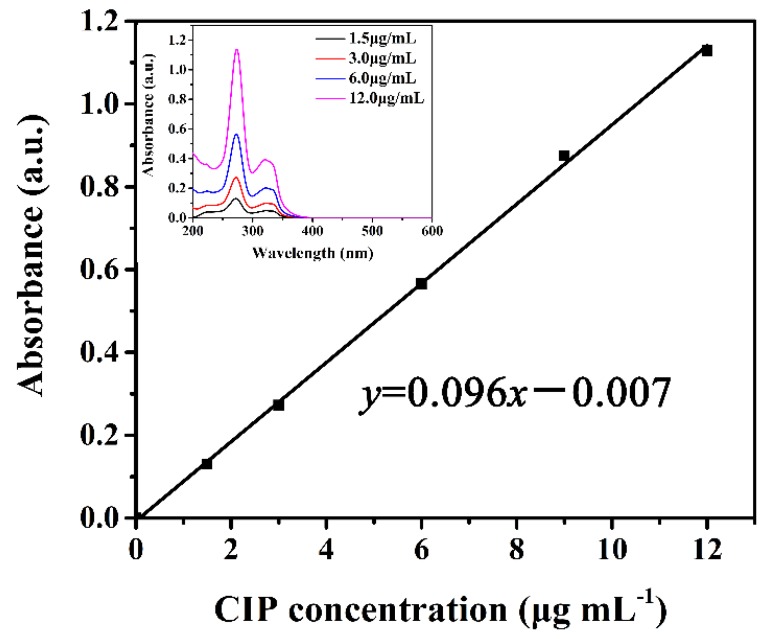
The UV absorbance of CIP as a function of varying CIP concentration.

We next quantitatively studied the loading efficiency (*L*%) of CIP into the PDA NCs. First, the UV-Vis intensity of CIP (Δ*I* = *I*_272_ − *I*_400_, *I_n_*: the intensity at the wavelength of *n* nm) was plotted as a function of predetermined concentration of CIP. Figure 5 illustrates that the value of Δ*I* (*y*) increases linearly with increasing concentration of CIP (*x*) (*R*^2^ = 0.99924), as in the following Equation (3):

(3) y=0.096x−0.007

Hence, the *L*% of CIP into the PDA NCs was 65.9%, which was calculated from the difference between the initial (*x*_0_) and equilibrated (*x*_1_) concentrations of the supernatant by using Equation (4):

(4) $$L\%=\left(1-\frac{x_{1}}{x_{0}}\right)\times 100\%$$

**Figure 6:**
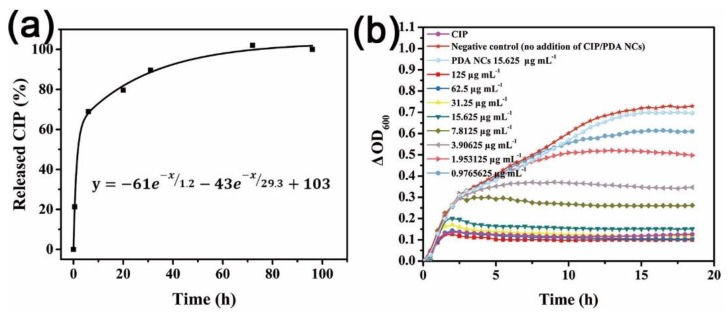
(**a**) CIP-release kinetics from PDA NCs at 37 °C in PBS; (**b**) Growth kinetics of the *E. coli* bacteria in the presence of CIP-PDA. A positive control (0.2 μg mL^−1^ pure CIP), a negative control (no addition of CIP/PDA NCs), and PDA NCs (15.625 μg mL^−1^) without the CIP were tested.

Under the same conditions, PEMA@PDA can only load 47.6% of CIP, suggesting CIP was loaded into the PDA NCs. When CIP-PDA was redispersed in DI water, CIP-PDA released exponentially the CIP moiety, as shown by monitoring the change in Δ*I* (Figure 6a). The release kinetics showed that approximately 50% CIP was unloaded after 6 h and leveled off until 70 h.

The antibacterial activity of CIP-PDA against Gram-negative *E. coli* (as a model bacterium) in Luria–Bertani (LB) media after 18 h of incubation was evaluated. The antibacterial kinetic growth assay was plotted by monitoring the change in the optical density at 600 nm (ΔOD_600_) of the specimens. Figure 6b shows that *E. coli* rapidly proliferated in the CIP-unloaded PDA NCs medium, resulting in an increase in ΔOD_600_. In comparison, the growth of *E. coli* in the presence of CIP-PDA was considerably decreased, depending on the incubation time and concentration of CIP-PDA. For example, when 3.90625 μg mL^−1^ CIP-PDA was incubated with *E. coli*, the change in ΔOD_600_ was inhibited after ~3 h of incubation. According to the *L*% and release kinetics fitting curve of CIP-PDA, we found the CIP moiety reached ~0.019 μg mL^−1^ after 18 h of continuous release. This value is close to the minimal inhibition concentration of CIP against *E. coli* (0.015 μg mL^−1^).

## 4. Conclusions

In summary, polymer assemblies that were prepared by a novel γ-ray-induced LLISA polymerization strategy showed spherical nanostructures with a diameter of ~300 nm. Next, PDA NCs were generated by interfacial polymerization of DA onto the polymer assemblies, followed by the removal of templates by using ethanol. PDA NCs were able to load and release CIP, such that the CIP-loaded PDA NCs inhibited the growth of *E. coli*.

## Supplementary Materials

The following are available online at https://www.mdpi.com/2073-4360/11/11/1754/s1, Figure S1: FTIR spectrum of PEMA; Figure S2: ^13^C NMR spectrum of PEMA; Figure S3: XPS spectra of PEMA; Figure S4: (**a**) DSC and (**b**) TG curves of PEMA; Figure S5: (**a**) SEM image, (**b**) ^1^H NMR spectrum (CDCl_3_), and (**c**) DSC curve of PMMA assembly; Figure S6: (**a**) SEM image, (**b**) ^1^H NMR spectrum (CDCl_3_), and (**c**) DSC curve of PBMA assembly; Figure S7: TG curves of specimens; Figure S8: (**a**) ^1^H NMR, (**b**) FTIR, and (**c**) solid-state ^13^C NMR spectra of PDA NCs.
