# Supplementary material for: Using γ-Ray Polymerization-Induced Assemblies to Synthesize Polydopamine Nanocapsules
Source: Polymers (Basel). 2019 Oct 25;11(11):1754. doi: 10.3390/polym11111754 (PMC6918355; doi:10.3390/polym11111754)
Supplement: Supplementary file 1 [file polymers-11-01754-s001.pdf]

polymers

MDPI

Supporting Information

Using γ-Ray Polymerization-Induced Assemblies to Synthesize Polydopamine Nanocapsules

Wenwen Jiang,[1,†] Xinyue Zhang,[2,†] Yafei Luan,[2] Rensheng Wang,[1] Hanzhou Liu,[1] Dan Li,[2,*] Liang Hu[1,*]

[1] School for Radiological and Interdisciplinary Sciences (RAD-X), Collaborative Innovation Center of Radiation Medicine of Jiangsu Higher Education Institutions and Jiangsu Provincial Key Laboratory of Radiation Medicine and Protection, Soochow University, Suzhou, Jiangsu, 215123, China.

[2] College of Chemistry, Chemical Engineering and Materials Science, Soochow University, Suzhou, Jiangsu, 215123, China.

† Both authors contributed equally to this work.

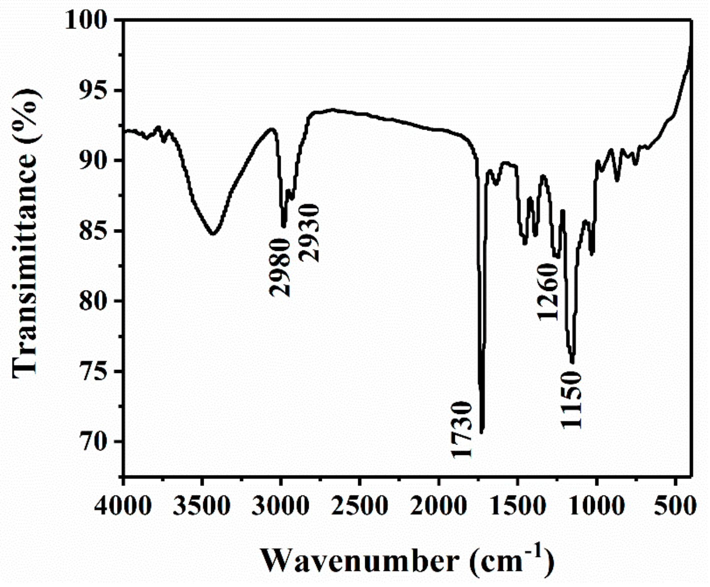

Figure 1. FTIR spectrum of PEMA.

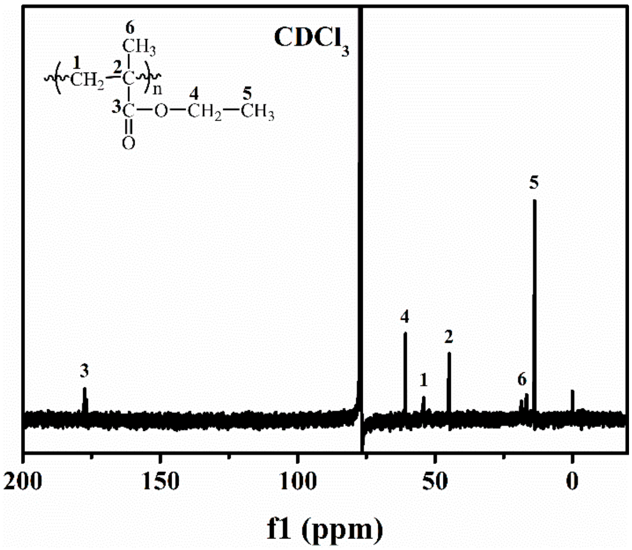

Figure 2. ^{13}C NMR spectrum of PEMA.

C 1s and O 1s can be detected at 285, 532 eV, respectively. The atomic percentage of C and O is 75%:25%. The O 1s peak can be divided into two peaks of C–O (533.1 eV) and C=O (531.7 eV) and the optimal ratio of the two peak areas is 1:1.

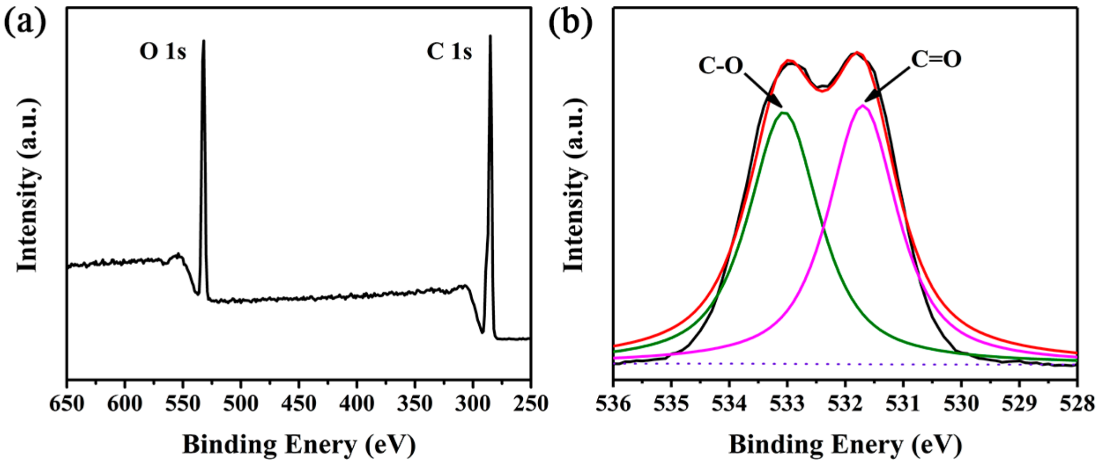

Figure 3. XPS spectra of PEMA.

PEMA has a T_g of ~61 °C. TG curve of PEMA shows an initial thermal decomposition temperature ($T_{5\%}$) of 193 °C and a residual carbon rate of 6%. Maximum weight loss rates occur at 260 and 370 °C.

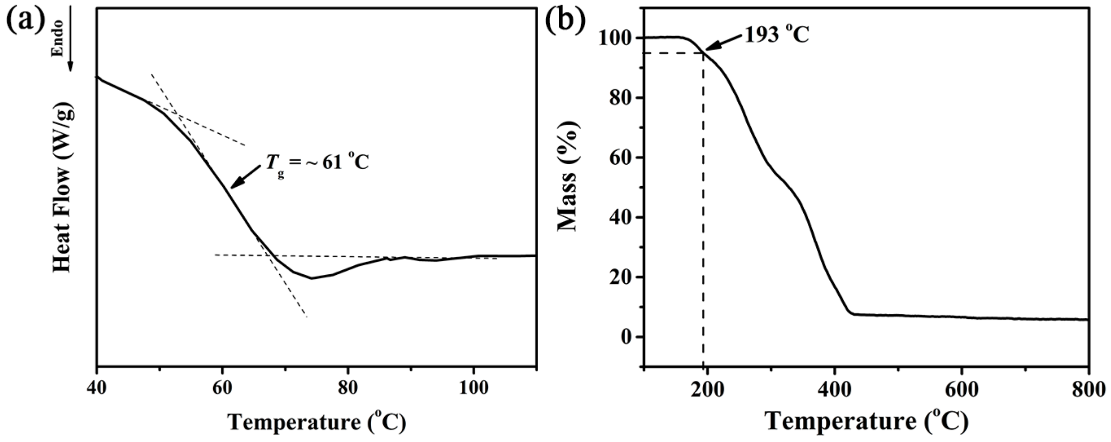

Figure 4. (**a**) DSC and (**b**) TG curves of PEMA.

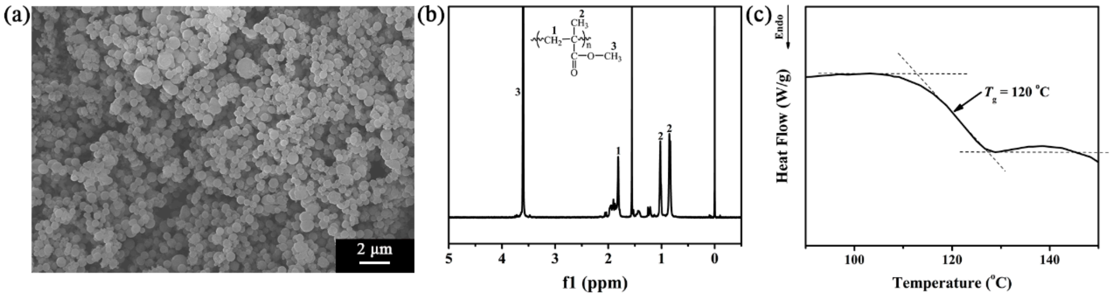

Figure 5. (**a**) SEM image, (**b**) ^{1}H NMR spectrum ($CDCl_3$), and (**c**) DSC curve of PMMA assembly.

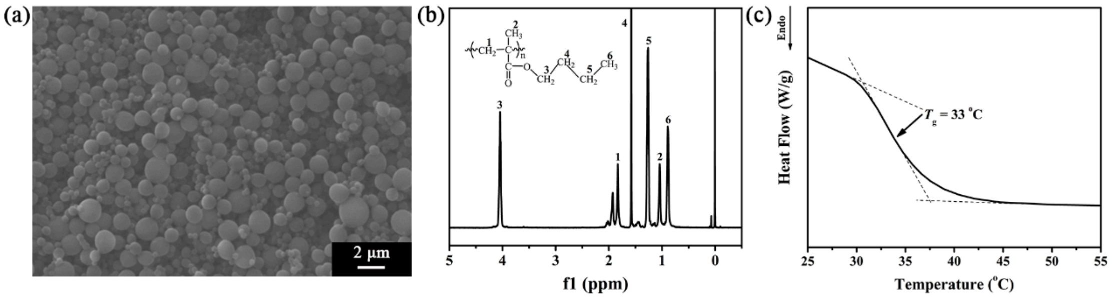

Figure 6. (a) SEM image, (b) ^{1}H NMR spectrum ($CDCl_3$), and (c) DSC curve of PBMA assembly.

Figure S7 shows that PEMA@PDA has three weightlessness platforms whose maximum weight loss rates correspond to the temperatures of 169, 275 and 390 °C, respectively, with $T_{5\%}$ of 225 °C and a residual carbon rate (a_2) of 18%. PDA NCs have two weightlessness platforms whose maximum weight loss rates correspond to the temperatures of 174, 376 °C, respectively, with $T_{5\%}$ of 225 °C and a residual carbon rate (a_3) of 60% Therefore, wt% PDA relative to PEMA (*R*%) in PEMA@PDA can be calculated using the equation:

$$R\% = \{[(1 - a_1) - (1 - a_2)]/[(1 - a_2) - (1 - a_1)]\} \times 100\%$$

where $a_1 = 6\%$ (data from Figure S4).

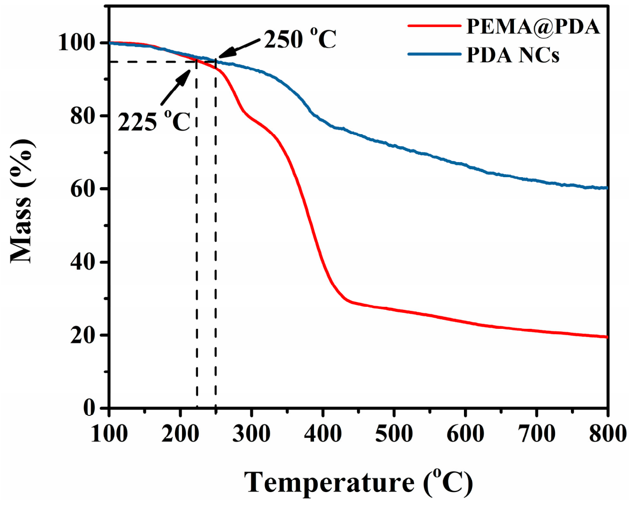

Figure 7. TG curves of specimens.

The signal at 4.04 ppm in (a) for –OC<u>*H*</u>$_2$CH$_3$ of PEMA disappeared. Furthermore, the intensity of C=O groups in (b) significantly decreased after the etch of PEMA assemblies. Solid-state ^{13}C NMR spectrum in (c) showed that the signal at 170, 145, 130–116 ppm can be assigned to C=O, C-OH and the aromatic carbon, respectively. The peak at 108 ppm is possibly due to quaternary bridgehead. The signals at 32 and 58 ppm can be assigned to the cyclized aliphatic carbon.

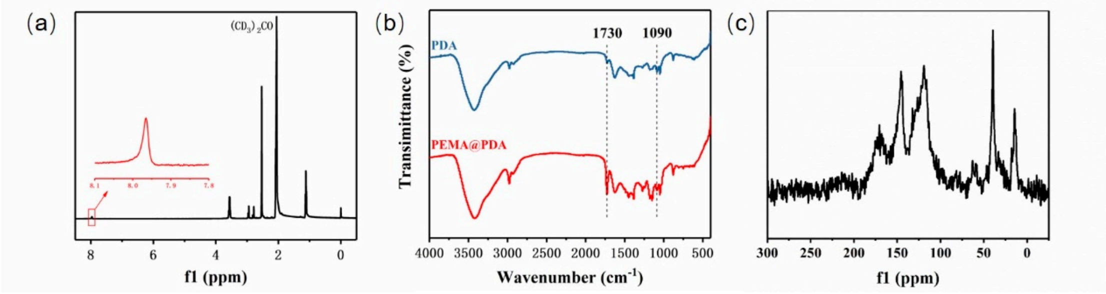

Figure 8. (**a**) ^{1}H NMR, (**b**) FTIR, and (**c**) solid-state ^{13}C NMR spectra of PDA NCs.
